# DMSO-free cryopreservation of hiPSC-derived cardiomyocytes: Low temperature characterization and protocol development

**DOI:** 10.21203/rs.3.rs-5183739/v1

**Published:** 2025-04-14

**Authors:** Akshat Satyanarayan Mallya, Tessa Burrows, Jeanne Hsieh, Troy Louwagie, James Dutton, Brenda Ogle, Allison Hubel

**Affiliations:** University of Minnesota Twin Cities; University of Minnesota Twin Cities; University of Minnesota Twin Cities; University of Minnesota Twin Cities; University of Minnesota Twin Cities; University of Minnesota Twin Cities; University of Minnesota Twin Cities

**Keywords:** Cardiomyocytes, cryopreservation, DMSO-free, Raman spectroscopy, NADES, protocol development

## Abstract

**Background:**

Human-induced pluripotent stem cell-derived cardiomyocytes (hiPSC-CMs) have attracted significant interest for use in disease modeling, drug discovery and potential therapeutic applications. However, conventional hiPSC-CM cryopreservation protocols largely use dimethyl sulfoxide (DMSO) as the cryoprotectant (CPA), which is linked with a loss of post-thaw recovery and function for various cell types and is not ideal for therapeutic protocols. Additionally, the effect of freezing parameters such as cooling rate and nucleation temperature on post-thaw recovery of hiPSC-CMs has not been explored.

**Methods:**

hiPSC-CMs were generated by Wnt pathway inhibition, followed by sodium I-lactate purification. Subsequently, biophysical characterization of the cells was performed. A differential evolution (DE) algorithm was utilized to determine the optimal composition of a mixture of a sugar, sugar alcohol and amino acid to replace DMSO as the CPA. The hiPSC-CMs were subjected to controlled-rate freezing at different cooling rates and nucleation temperatures. The optimum freezing parameters were identified by post-thaw recoveries and the partitioning ratio obtained from low temperature Raman spectroscopy studies. The post-thaw osmotic behavior of hiPSC-CMs was studied by measuring diameter of cells resuspended in the isotonic culture medium over time. Immunocytochemistry and calcium transient studies were performed to evaluate post-thaw function.

**Results:**

hiPSC-CMs were found to be slightly larger than hiPSCs and exhibited a large osmotically inactive volume. The best-performing DMSO-free solutions enabled post-thaw recoveries over 90%, which was significantly greater than DMSO (69.4 ± 6.4%). A rapid cooling rate of 5°C/min and a low nucleation temperature of −8°C was found to be optimal for hiPSC-CMs. hiPSC-CMs displayed anomalous osmotic behavior post-thaw, dropping sharply in volume after resuspension. Post-thaw function was preserved when hiPSC-CMs were frozen with the best-performing DMSO-free CPA or DMSO and the cells displayed similar cardiac markers pre-freeze and post-thaw.

**Conclusions:**

It was shown that a CPA cocktail of naturally-occurring osmolytes could effectively replace DMSO for preserving hiPSC-CMs while preserving morphology and function. Understanding the anomalous osmotic behavior and managing the excessive dehydration of hiPSC-CMs could be crucial to improve post-thaw outcomes. Effective DMSO-free cryopreservation would accelerate the development of drug discovery and therapeutic applications of hiPSC-CMs.

## Background

Cardiovascular diseases are among the leading causes of morbidity and mortality globally, with ischemic heart diseases (IHDs) alone accounting for approximately 9 million deaths annually [1]. This number is expected to continue to rise due to aging populations [2]. The heart has very limited regenerative potential [3], and this effect is not sufficient to replace the large portions of cardiac tissue affected in IHDs. Transplantation of cardiomyocytes into infarcted hearts has emerged as a potential promising treatment, with improvement of cardiac function observed in animal studies [4–6].

Particularly, cardiomyocytes derived from human-induced pluripotent stem cells (hiPSCs), referred to as hiPSC-CMs, have garnered significant interest. This interest is due to the exceptional self-renewal capability of hiPSCs, which is crucial for regenerative therapy, disease modeling and drug discovery applications [7–11], where large quantities of cardiomyocytes are desired. Moreover, a mounting body of evidence has suggested that hiPSC-CMs can effectively emulate native cardiomyocytes in transplantation [12] and drug discovery [13–15] applications. Effective cryopreservation of hiPSC-CMs is essential to improve their accessibility to patients and researchers by greatly decreasing manufacturing and supply chain costs. Furthermore, the development of an optimized hiPSC-CM cryopreservation protocol would support the creation of biobanks, from which cells could be withdrawn whenever desired.

However, efforts to cryopreserve cardiomyocytes have met with limited success. Most studies report relatively low post-thaw viabilities between 50 and 80% [16–18] with changes observed in the post-thaw function of the cardiomyocytes such as reduced contractility and increased susceptibility to drug-induced arrhythmic events [17]. All of the conventional cardiomyocyte cryopreservation protocols utilize dimethyl sulfoxide (DMSO), typically at a concentration of 10%, as one of the components of the cryoprotective agent (CPA). Despite its widespread use, in vitro studies have shown DMSO to cause cell death and compromise the cell membrane due to its permeabilizing properties [19]. Additionally, patients receiving DMSO infusions report various adverse allergic, gastrointestinal, neurological and cardiac side effects [20–23]. Moreover, DMSO is associated with epigenetic effects such as disruptions in DNA methylation mechanisms [24, 25], which makes it particularly problematic for use with hiPSC-derived cells. Thus, DMSO needs to be washed out before infusion, which leads to additional cell loss post-thaw. Lastly, 10% DMSO has also been shown to damage and leach contaminants from plasticware [26], which can lead to manufacturing challenges.

Consequently, there is a need to develop DMSO-free cryopreservation methods for hiPSC-CMs. To that end, we have demonstrated in previous studies that combinations of naturally-occurring osmolytes can effectively preserve a variety of cell types, ranging from T-lymphocytes [27, 28, 30] to mesenchymal stem cells [29, 30] and hiPSCs [31]. Yet, unlike the one-size-fits-all nature of DMSO, it has been demonstrated that specific combinations of concentrations lead to better post-thaw outcomes [27, 30, 31], which requires CPA optimization for a specific cell type. In addition to CPA optimization, the optimal freezing parameters also need to be identified for hiPSC-CMs. Freezing parameters such as cooling rate [34] and nucleation temperature [35] have a significant effect on the post-thaw recovery of most cell types. However, the effect of these freezing parameters on hiPSC-CM recovery has not been explored. In the existing literature, when controlled-rate freezing has been performed, the freezing protocols have usually not been specified [32]. When specified, a cooling rate of 1°C/min has been chosen for most of the freezing process [16, 17], with no clear consensus on the optimal cooling rate [33]. Moreover, the impact of nucleation temperature on hiPSC-CM cryopreservation has not been explored.

In this study, we determined biophysical characteristics of interest both pre-freeze and post-thaw. A differential evolution (DE) algorithm was used to optimize a DMSO-free CPA composition for hiPSC-CMs to maximize post-thaw recovery. Further, we also assessed the effect of cooling rate and nucleation temperature on the recovery of hiPSC-CMs. The effect of cooling rate was further explored by performing low temperature Raman spectroscopy and analyzing the solute partitioning of hiPSC-CMs. These results were used to identify the optimal controlled rate freezing parameters for hiPSC-CMs. Finally, the post-thaw function of the hiPSC-CMs was measured by performing calcium transient studies. These studies will advance our basic understanding of the freezing behavior of cardiomyocytes and help us to preserve this important cell type more effectively.

## Methods

### Cardiomyocyte differentiation

The CCND2 hiPSC line (provided by Dr. Jianyi Zhang, University of Alabama at Birmingham), previously reprogrammed from cardiac fibroblasts of a healthy female donor [56] and genetically modified to overexpress cyclin D2 to enhance cardiac differentiation yield [36, 37], was used in this study. The hiPSCs were cultured as adherent colonies on 6-well plates coated with Matrigel (Corning) and fed with mTeSR1 medium (STEMCELL Technologies) for 5 days to reach 80–90% confluency. hiPSCs used in this investigation were between passage 57 and 79. To harvest the hiPSCs, the culture medium was removed, and each well was washed once with sterile DPBS (Gibco) before adding 1 ml of room-temperature Accutase (STEMCELL Technologies). The plates were incubated at 37°C for 8 minutes. To stop the Accutase reaction, 0.5 ml of mTeSR 1 medium was added to each well. The singularized cells were collected, centrifuged at 200g for 5 minutes to remove the Accutase solution, and resuspended in mTeSR1 with 5 μM ROCK inhibitor (Y27632). The hiPSCs were seeded at a density of 1×10^6^ cells/well on Matrigel-coated 12-well plates and maintained in a 5% CO2 incubator at 37°C for 24 hours. The hiPSC cultures were routinely tested for mycoplasma contamination.

The hiPSCs were then subjected to a fully defined direct cardiac differentiation protocol by modulating Wnt/β-catenin signaling. On Day −1, 1 ml of fresh mTeSR1 medium was added to each well. On Day 0, cardiac differentiation was initiated on 100% confluent hiPSCs by adding 6.5 μM GSK3-β inhibitor (CHIR99021) in 2 ml of RPMI/B-27 without insulin (RPMI minus) medium into each well for 48 hours to induce mesoderm formation. On Day 2, the CHIR99021 medium was replaced with fresh RPMI minus medium supplemented with 5 μM Wnt inhibitor (IWP2) to induce cardiac differentiation. On Days 4 and 6, fresh RPMI minus medium was added to each well. On Day 8 and every 3 days thereafter, fresh maintenance medium (RPMI/B-27) was added. Spontaneous twitching was observed on Day 7, and robust spontaneous contraction was found to occur by Day 12.

Next, cardiomyocyte enrichment was performed on Days 10–14 by adding 2 mL DMEM (without glucose) with 4 mM sodium L-lactate every 2 days to achieve a cardiomyocyte population purity of > 98% [37]. On Days 14–16, cells were maintained in RPMI/B-27 medium (RPMI+). Day 20 hiPSC-CMs were harvested by treating the wells with 0.25% Trypsin-EDTA for 12 minutes at 37°C. The singularized hiPSC-CMs were resuspended in RPMI/B-27 medium with 20% fetal bovine serum (RPMI20) and 5 μM ROCK inhibitor (Y27632) and allowed to recover for 30 minutes before being used for all of the experiments performed in this study.

### Cryoprotectant formulation

Naturally-occurring osmolytes such as trehalose (Sigma-Aldrich), glycerol (Humco) and isoleucine (Sigma-Aldrich) were was used to formulate the DMSO-free CPA. Both the DMSO and DMSO-free CPAs utilized an isotonic basal buffer of Normosol R. A 10% concentration solution of DMSO (Sigma-Aldrich) in Normosol R was taken as the control CPA for all studies.

### Controlled rate freezing

Singularized cardiomyocytes were resuspended in Normosol R at a concentration of 6 to 8 million cells per ml and gently pipetted to break any clumps. 0.5 ml of the cell suspension was transferred to 1.8 ml cryovials (Nunc CryoTubes, Thermo Scientific) and twice the working concentration of the CPA solution was added dropwise in increments of 150 μl,150 μl and 200 μl to reach a final volume of 1 ml. The DMSO and DMSO-free vials were left loosely capped at room temperature for 30 min and 1 hour, respectively to allow the CPA to internalize sufficiently. A “dummy” vial containing 10% DMSO in Normosol R was prepared, with a type T thermocouple inserted through a hole in the cap to monitor the internal temperature. Next, the vials were tightly capped and transferred to a controlled rate freezer (Kryo 560 – 16, Planer) to undergo freezing according to the following protocol.

1. Starting temperature of 4°C.
2. Ramp at −10°C/min to 0°C.
3. Hold at 0°C for 10 min to allow the temperature inside (sample temperature) and outside (chamber temperature) the vials to equilibrate.
4. Ramp at - *CR*°C/min to *T*_*NUC*_.
5. Hold at *T*_*NUC*_ for 15 min to allow the temperature inside and outside the vials to equilibrate.
6. Ramp at - *CR*°C/min to −60°C.
7. Ramp at −10°C/min to −100°C.

Where *CR* and *T*_*NUC*_ were the cooling rate and the nucleation temperature, respectively. At the end of step 5, the vials were briefly sprayed with a jet of liquid nitrogen using a cryogun (Brymill) to induce nucleation at the desired nucleation temperature. After the completion of the freezing protocol, the vials were immediately transported to a liquid nitrogen dewar and stored in the vapor phase of liquid nitrogen.

### Thawing

After retrieving the vials from the vapor phase of liquid nitrogen, the vials were thawed by swirling them in a 37 °C water bath for around 2.5 min until most visible ice melted. Next, the vials were moved to a biosafety cabinet and pipetted up and down gently to ensure an even distribution for enumeration. To measure the immediate viability, a small cell sample of 50 μl was mixed with acridine orange/propidium iodide (AO/PI) to quantify viability based on membrane integrity. The number of live and dead cells was counted and the size distribution of the cells was obtained using an automated cell counter (Countess 3 FL, Thermo Fisher). Post-thaw recovery was evaluated using the formula in Eq. 1.


#(1) $$Recovery=\frac{\;Number\;of\;live\;cells\;post\;thaw\;}{\;Number\;of\;live\;cells\;pre\;freeze\;}$$

After counting, the contents of the vials were transferred to centrifuge tubes and 1 ml of RPMI20 was slowly added dropwise to each tube. The tubes were allowed to rest for 10 min to minimize osmotic shocks. After that, RPMI20 was added dropwise with periodic shaking after every 1 ml until a final volume of 10 ml was reached. The tubes were centrifuged at 200g for 5 min, resuspended in RPMI + with 1 μM ROCK inhibitor and plated onto 12-well plates coated with Matrigel.

### DE Algorithm

A DE algorithm with the basic mutation strategy (DE/1/rand/bin) was used to rapidly optimize the DMSO-free CPA without testing compositions over the entire parameter space. The post-thaw recovery of the cardiomyocytes was chosen as the functional metric of the algorithm. The parameter space for the algorithm was determined by taking the experimental cytotoxicity limit for a particular component from previous studies [30, 31] and dividing it into ten levels. The components of the DMSO-free CPA (trehalose, glycerol and isoleucine) were chosen based on previous studies which had demonstrated comparative or better cryopreservation outcomes using combinations of sugars, sugar alcohols and amino acids when compared to DMSO [28, 29].

The initial compositions to be tested (Generation 0) were determined by a direct stochastic search and freezing and thawing was performed to determine the post-thaw recovery for the selected compositions. The recovery results were used by the algorithm to generate mutated versions of Generation 0 to be tested. The compositions yielding the best recoveries from the mutation and the current generation were outputted as the emergent population (Generation 1). This cycle was repeated until convergence was achieved; that is, when the emergent population was identical to the previous generation. This convergence condition has been demonstrated to identify the global maximum with over 95% accuracy [31]. For this study, the DE algorithm was programmed with a mutation rate of 0.85 and a crossover of 1. The population size, or the unique number of compositions outputted per generation, was set to 9. The cells were frozen via controlled rate freezing at a cooling rate of 1°C/min with nucleation induced manually at −4°C for all generations.

### Low temperature Raman spectroscopy

A Peltier stage (Thermonamic Electronics) and a series 800 temperature controller (Alpha Omega Instruments) were used to freeze samples of cardiomyocytes in 10% DMSO. Cooling rates of 1°C/min, 3°C/min and 5°C/min were tested and ice nucleation was induced at approximately − 8°C by briefly bringing the sample in contact with a liquid-nitrogen-chilled probe. The final temperature was held at a stable temperature of −50°C, at which point Raman data were collected. Raman spectroscopic measurements were made using the WiTec Confocal Raman Micrscope Alpha 300R with UHTS spectrometer and DV401 CCD detector with 600/mm grating. A 532nm Nd:YAG laser was used as the excitation source. A 100x air objective (NA 0.90, Nikon Instruments) was used to focus the laser. The size of each pixel in the field of view was 333 × 333 nm, and Raman spectra were obtained at each pixel with a 0.2 s integration time.

Intensities of Raman signals and boundaries of cells were measured using similar techniques from previously defined methods [44]. Briefly, Raman spectra were acquired at each pixel, and different molecular targets were used to create heat maps of cells, DMSO, and ice (Table 1). Raman heat maps of components of interest were generated by discrete trapezoidal area integration (MATLAB) of various signal bonds. Cell boundaries were identified for the different cooling rates using a combination of the amide I signal (C = C stretching) and oxymyoglobin (oMb) to form an outline of the cell boundary. The region not occupied by ice or cells was identified by the symmetric C-S stretching in DMSO, and the ice region was identified by its O-H stretching.

The partitioning ratio (Eq. 2) was calculated as the intensity of DMSO outside of the cell in the non-ice region divided by the intensity of DMSO inside of the cell. The intensity is proportional to the concentration of DMSO in each compartment.


#(2) $$\;Partitioning\;ratio\;=P=\frac{C_{o}}{C_{i}}$$

Where $C_{o}$ and $C_{i}$ represent the concentrations of the CPA outside and inside the cell, respectively.

### Immunocytochemistry

Cardiomyocytes plated onto Matrigel-coated 12-well plates were fixed using formalin and permeabilized using a solution of 0.2% Tween 20 in dPBS. The permeabilized cardiomyocytes were incubated with anti-Actin (1:5000, Sigma-Aldrich, A2103) and Nkx-2.5 (1:200, Santa Cruz Biotechnology, sc-376565) antibodies to stain F-actin and the Nkx2.5 transcription factor, respectively and counterstained with DAPI (Roche). After 24 hours, the primary antibodies were aspirated and the corresponding fluorescent secondary antibodies (Invitrogen) were added to detect primary antibody binding. Stained cultures known to not express the antigens of interest and unstained cardiomyocyte cultures were used as negative controls. The stained cells were imaged using a Leica DMI6000B microscope using a 20x air objective and a DFC365FX camera.

### Calcium transient study

To assess calcium transient measurements, singularized hiPSC-CMs were re-plated to form a monolayer. The cell suspension was transferred to a conical tube containing RPMI20 at twice the volume of the cell suspension and incubated for 30 minutes. The suspension was then centrifuged at 200 g for 5 minutes to remove the supernatant. The hiPSC-CMs were resuspended in RPMI + medium with 10 μM ROCK inhibitor and seeded at a density of 2e6 cells/well on 12-well plates. The re-plating medium was replaced after 24 hours with fresh RPMI+. The hiPSC-CMs were fed with fresh RPMI + every 2 days until a connected monolayer was formed for calcium transient measurement.

Calcium ion movement was assessed using a DMi8 fluorescence microscope (Leica, Wetzlar, Germany) and LAS X software. The hiPSC-CM monolayer was incubated with 5 μM Fluo-4 acetoxymethyl ester (Fluo-4 AM) per 1 mL of culture medium at 37°C for 30 minutes, followed by an exchange to Tyrode’s salt solution for another 30-minute incubation at 37°C. Subsequently, the hiPSC-CMs were placed onto the microscope stage and covered with a heating plate to maintain the temperature at 37°C. Fluo-4 AM intensity was recorded at a frame rate of 6.90 Hz with a 30 ms exposure time. The acquired data was processed using ImageJ to obtain a time trace of the calcium signal and analyzed with a custom-written Python script to extract maximal and minimal intensity and corresponding time points for each peak. Peak amplitude was determined by F/F_0_, where F = < F_max_> - <F_min_> and F_0_ = < F_min_>. <F_max_> and < F_min_> represent the averaged maximum and minimum intensity for each peak, respectively.

### Statistical analysis

Power analysis was performed to ensure an adequate sample size to achieve a power of 0.95. Statistical validation was performed using two-sample t-tests with a 95% confidence level, and the null hypothesis was rejected for p < 0.05. Error bars in all figures represent the standard error.

## Results

### Biophysical properties of hiPSC-CMs

**Figure 1:**
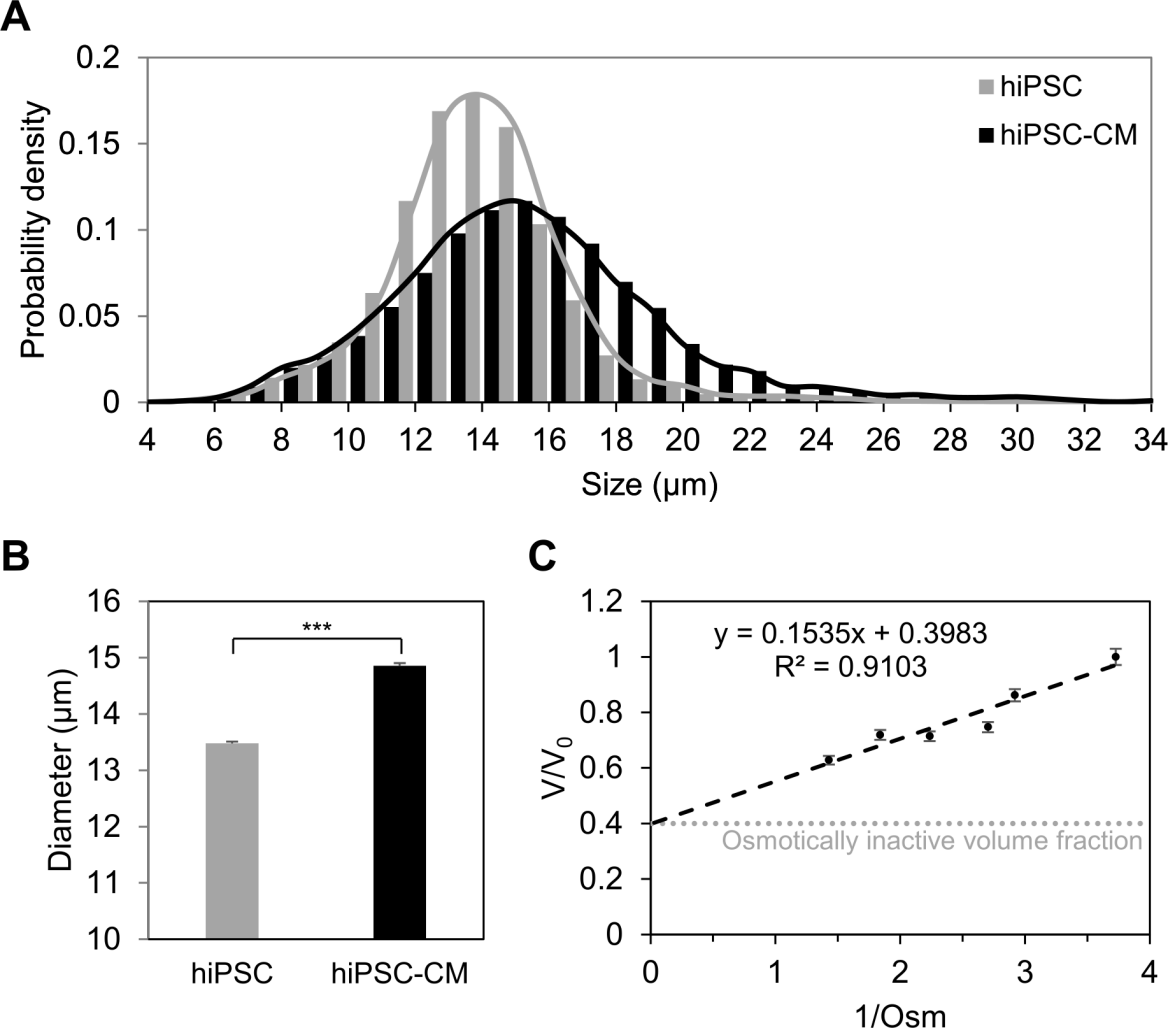
Biophysical properties of hiPSC-CMs. (A) Histogram of the size distribution of hiPSCs and hiPSC-CMs obtained from the Countess 3 FL cell size data. (B) Average diameter of hiPSCs (n = 7173) vs hiPSC-CMs (n = 7455). The difference in cell diameters was statistically significant. (***, p < 0.001) (C) Boyle-van’t Hoff plot of hiPSC-CMs displaying the variation of normalized cell volume fraction (V/V_0_) as a function the solution osmolarity (1/0sm). Here, V and V_0_ represent the volume of the cell in a solution of a given osmolarity and the volume of the cell in an isotonic medium, respectively. The osmotically inactive volume fraction of hiPSC-CMs is denoted by the dotted gray line. The error bars represent the standard error.

Initial studies involved basic biophysical characterization of the cells. The histogram of size distribution (Fig. 1A) indicated that the variation in size of hiPSC-CMs (standard deviation = 4.11 μm) was greater than that of hiPSCs (standard deviation = 2.74 μm), as evident by the long tails of the hiPSC-CM histogram. Additionally, it was noted that hiPSC-CMs in suspension were 14.86 ± 0.05 μm in diameter (Fig. 1B), which was slightly greater than the size of hiPSCs (13.48 ± 0.03 μm) in suspension. The osmotically inactive cell volume fraction for the cells was also determined. hiPSC-CMs were suspended in sucrose solutions of differing osmolarities and the cell diameter determined. This data was graphed in the form of a Boyle-van’t Hoff plot (see Fig. 1C). The osmotically inactive cell volume fraction for hiPSC-CMs was 0.4.

### DE Algorithm-Driven DMSO-free CPA Optimization

**Figure 2:**
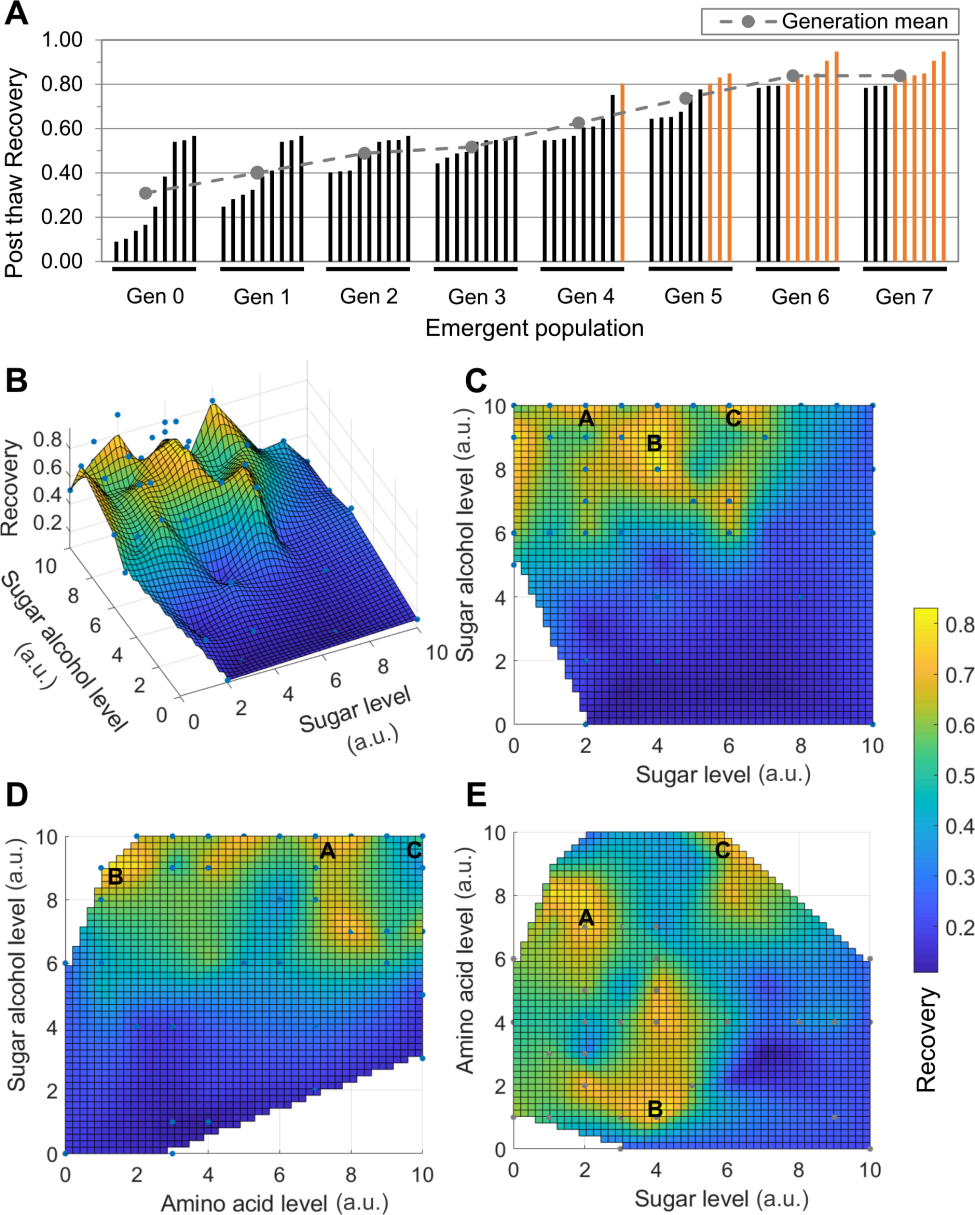
DE algorithm-driven DMSO-free CPA optimization results of hiPSC-CMs. (A) Post-thaw recovery for each generation (Gen) of the DE algorithm. Each bar within a generation represents the recovery (Equation 1) of one of the compositions of that generation. Mean post-thaw recovery of each generation is denoted by a gray circle. (B) 3D isometric view of the sugar-sugar alcohol recovery contour. (C-E) Top-down views of the (C) sugar-sugar alcohol, (D) sugar alcohol-amino acid and (E) sugar-amino acid recovery contours. Level 0 corresponds to the absence of the component. Concentrations of components corresponding to level 10 for the sugar, sugar alcohol and amino acid were set to values determined from previous studies. [30, 31]

The post-thaw recovery of cells for a given population vector (i.e. array of solution compositions) is given in Fig. 2A. It was observed that the post-thaw recovery increased with increasing generations. Post-thaw recoveries shown in orange are greater than 80%. Beyond Generation 6, there were no new improved compositions discovered, which signaled that convergence was achieved and led to the termination of the algorithm. The post-thaw recovery for the three top solution compositions exceeded 90%. In comparison, the average recovery of the DMSO controls across the different generations was 69.4 ± 6.4%.

Recovery contours (Fig. 2B-E) were generated by plotting the recovery points for each tested composition and performing natural neighbor interpolation within the convex hull of tested points. Duplicate recovery points for the same composition were averaged for creating the contours. From the Sugar-Sugar Alcohol recovery contour (Fig. 2B), it was observed that there are clear regions where post-thaw recovery increases and decreases rapidly with changes in composition creating islands of high post-thaw recovery. Despite the non-linear nature of the contours, the top-down view of the Sugar-Sugar Alcohol and Sugar Alcohol-Amino Acid recovery contours (Fig. 2C-D) clearly showed that increasing concentrations of glycerol correspond to an increase in recovery. On the other hand, the effect of trehalose and isoleucine concentration on recovery was not linear. Instead, it was found that specific combinations of trehalose and isoleucine concentrations create recovery hotspots (Fig. 2E). The three best-performing solutions from the DE algorithm, solutions A, B and C in increasing order of sugar level, are marked on the recovery contours.

### Cooling Rate Optimization

**Figure 3:**
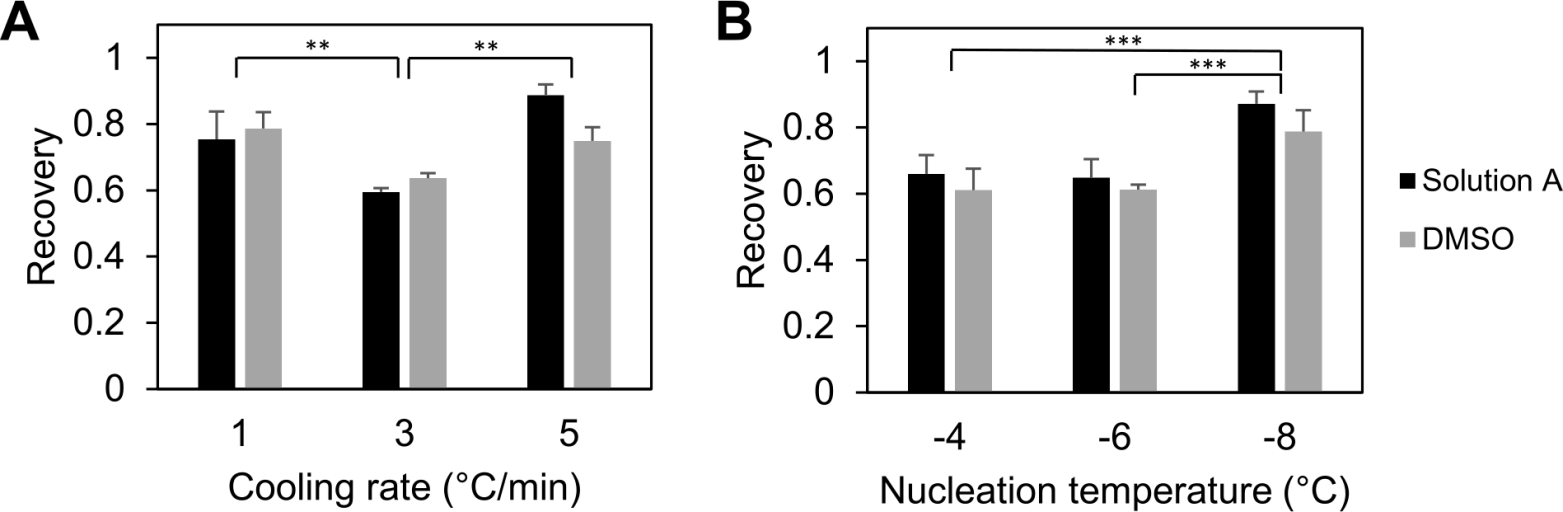
HiPSC-CM controlled-rate freezing parameter optimization. (A) Post-thaw recovery vs cooling rate for hiPSC-CMs. (B) Post-thaw recovery vs nucleation temperature for hiPSC-CMs. n = 4 for each condition and each CPA (Solution A or 10% DMSO). The error bars represent the standard error. (**, p < 0.01, ***, p < 0.001)

The post-thaw recovery of hiPSC-CMs frozen at cooling rates of 1,3 and 5°C/min was determined using a nucleation temperature of −4°C. As shown in Fig. 3 A, it was observed that the recovery was significantly higher for the 1 and 5°C/min cooling rates when compared to 3°C/min for hiPSC-CM cryopreserved in DMSO-free solutions as well as 10% DMSO controls. However, there was no significant difference in the immediate post-thaw recoveries of 1 vs 5°C/min. To determine the optimum cooling rate, the hiPSC-CMs were seeded at a constant density of 1×10^6^ cells per ml on Matrigel-coated 12 well plates and the reattachment and function was tracked for 1 week. Despite having largely similar post-thaw recoveries, it was found that the hiPSC-CMs frozen at 5°C/min consistently started beating earlier and had a larger number of beating cells when compared to the cells frozen at 1°C/min. From these results, it was determined that a cooling rate of 5°C/min is optimal for the preservation of hiPSC-CMs.

### Nucleation Temperature Optimization

Three batches of hiPSC-CMs were frozen at the previously determined optimal cooling rate of 5°C/min with manual nucleation induced at −4°C, −6°C and − 8°C. The post-thaw recoveries for the three cases are displayed in Fig. 3B. While there was no significant difference in the post-thaw recoveries of cells frozen with nucleation at −4°C and − 6°C, it is evident from the data that the hiPSC-CMs frozen with nucleation induced at −8°C recorded a significantly higher post-thaw recovery. Monitoring post-thaw attachment and function after seeding on Matrigel-coated 12 well plates further reinforced the findings, with a larger number of cells attaching and beating for the − 8°C condition. These findings indicated that the induction of ice nucleation at −8°C while freezing hiPSC-CMs lead to the most favorable post-thaw outcomes.

### Low temperature Raman spectroscopy

**Figure 4:**
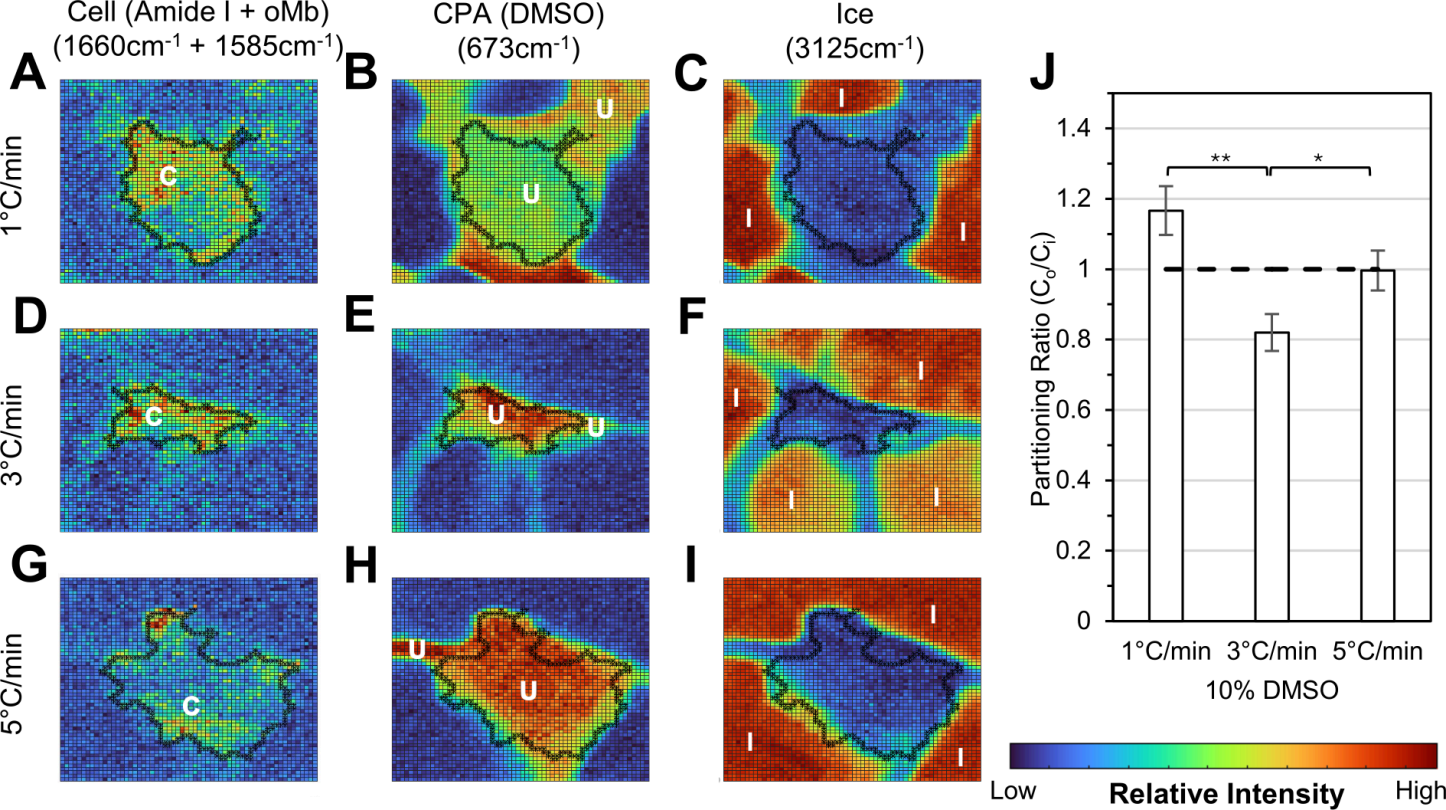
Relative Raman spectroscopy signals of cardiomyocyte cells at −50°C. Representative images of cardiomyocytes frozen to −50°C at varying cooling rates. Heatmaps corresponding to specific signals (Table 1) are of (A,D, and G) amide I and oMb combined representing the location of the cell marked by symbol C, (B, E, and H) DMSO representing the unfrozen section marked by symbol U, and (C, F, and I) ice marked by symbol I for cardiomyocytes with cooling rates of 1°C/min, 3°C/min and 5°C/min, respectively. (J) The partitioning ratio (Equation 2) is shown for each cooling rate condition. Each Raman figure was taken within a 60×60 pixel area. The scanning area for each image is 20×20 μm. Relative signal intensity increases from blue to red. The error bars represent the standard error. (*, p < 0.05, **, p < 0.01)

Raman spectroscopy was used to investigate three different cooling rates: 1°C/min, 3°C/min, and 5°C/min. The cell boundaries were identified, and partitioning ratios were calculated as described in the methods section in order to understand the distribution of cryoprotective agents for different freezing conditions. Cells frozen with a cooling rate of 1°C/min (Fig. 4A-C) had a partitioning ratio of 1.16 ± 0.19, cells frozen with a cooling rate of 3°C/min (Fig. 4D-F) had a partitioning ratio of 0.82 ± 0.13, and cells frozen with a cooling rate of 5°C/min (Fig. 4G-I) had a partitioning ratio of 1.00 ± 0.14. No significant intracellular ice formation was observed at any of the cooling rates.

The partitioning ratio of cells with a 3°C/min cooling rate were statistically significantly lower compared to cells cooled at 1°C/min and 5°C/min. This result is consistent with the result given in Fig. 3A demonstrating that post-thaw recovery at 3°C/min is less than that at 1 and 5°C/min.

### Post-thaw osmotic behavior

**Figure 5:**
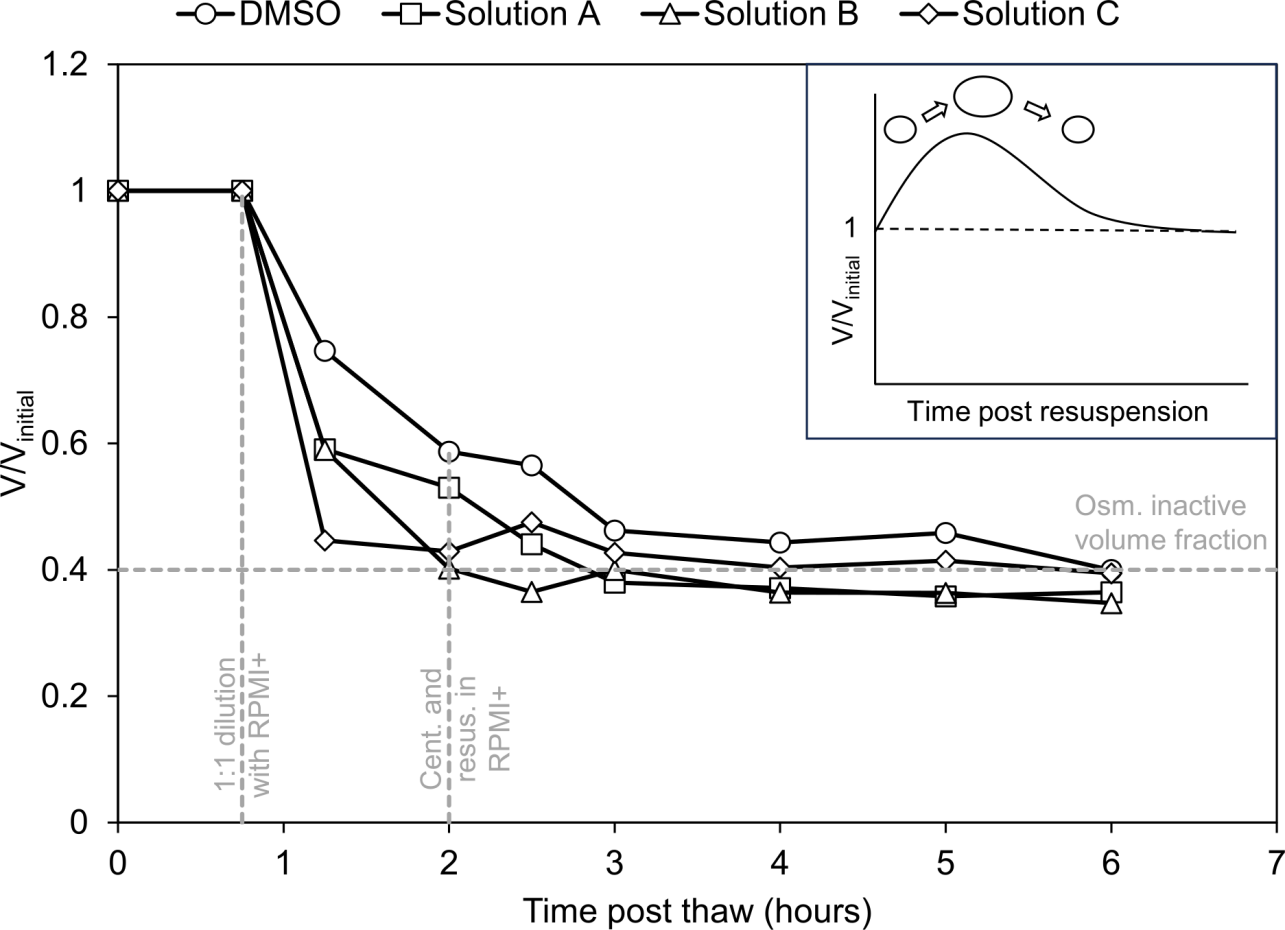
Post-thaw osmotic behavior of hiPSC-CMs. Each data point represents the normalized cell volume fraction (V/V_intial_) derived from the mean size of the hiPSC-CMs (obtained from the Countess 3 FL imaging data). Here V and V_initial_ represent the volume of the cell at a given time point and the volume of the cell before the addition of the culture medium. n > 200 for each CPA at each time point. At 0.75 hours post-thaw, corresponding to the first vertical dashed line from the left, hiPSC-CMs were diluted 1:1 with RPMI+. At 2 hours post-thaw (second vertical dashed line) the cells were centrifuged and resuspended in RPMI+. The inset represents the expected normalized cell volume curve for stem cells [55].

On addition of the culture medium to hiPSC-CMs in the CPA post-thaw, it was noticed that the hiPSC-CMs displayed an anomalous osmotic behavior post-thaw when compared to other cell types. As displayed in Fig. 5, this was characterized by the absence of swelling and a steep drop in the cell volume post-thaw. For the DMSO-free best performing CPA solutions, it was noted that the cell volume almost dropped to the previously determined osmotically inactive volume fraction of 0.4 within the first 30 mins of culture media addition. For DMSO, the overall trend of cell volume over time was similar, however, the drop in cell volume was gentler.

### Post-thaw assessment

**Figure 6:**
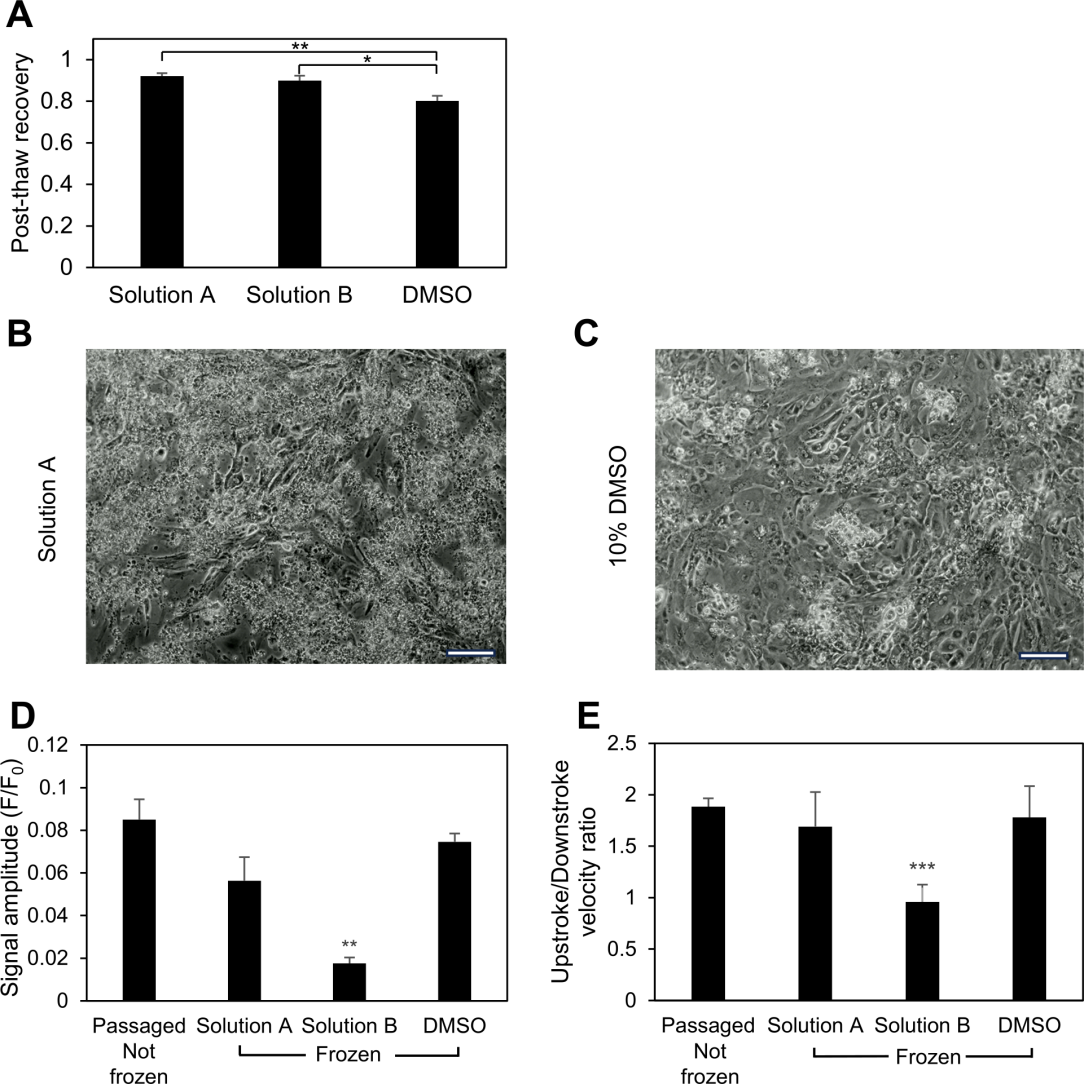
Post-thaw functional assessment of hiPSC-CMs. (A) Post-thaw recovery for hiPSC-CMs frozen at the optimal freezing parameters using the two best performing DMSO-free CPAs and 10% DMSO. (B-C) HiPSC-CMs frozen using (B) Solution A and (C) 10% DMSO replated post-thaw and cultured for 1 week. Scale bar: 100μm (D-E) Calcium transient analysis of hiPSC-CMs to find the (D) signal amplitude described by the ratio of the difference between the averaged maximum and minimum intensity of calcium transient peak and the averaged minimum intensity of the calcium transient peak (F/F_0_), and the (E) ratio of the upstroke velocity to downstroke velocity, of hiPSC-CMs frozen using DMSO-free Solution A and B and 10% DMSO compared to unfrozen cells which were detached and replated. n = 7 for unfrozen cells, n = 5 for Solution A, n = 3 for Solution B and 10% DMSO. The error bars represent the standard error. (**, p < 0.01, ***, p < 0.001)

The next phase of the investigation involved determining the post-thaw phenotype and function of the cells. HiPSC-CMs were frozen at the optimum cooling rate and nucleation temperature of 5°C/min and − 8°C, respectively using the two best-performing DMSO-free CPAs and the 10% DMSO control. As shown in Fig. 6A, it was found that the post-thaw recoveries of the DMSO-free best performers were significantly greater than that of DMSO (80.19 ± 4.22%), with Solution A recording the highest recovery (92.06 ± 2.50%). Also, it was noted that there was no significant difference in the recoveries of the two best-performing DMSO-free CPAs.

The contractile function of the cells is important and transient variations in calcium flux in the cells is commonly used as a surrogate of that function. As a result, calcium transient studies of the frozen and thawed hiPSC-CMs were performed and compared to unfrozen passaged and replated cells to check if post-thaw function was preserved. The calcium signal amplitude of the DMSO-free Solution A and DMSO were not significantly different from the unfrozen replated cells (see Fig. 6D). However, Solution B exhibited a marked reduction in signal amplitude compared to the other cases. Similarly, as indicated by Fig. 6E, the ratio of upstroke velocity to downstroke velocity showed no significant difference among Solution A, DMSO, and the freshly re-plated control, suggesting that the shape of the calcium transient trace was not affected by the preservation process when using Solution A or DMSO. Consistent with the trend of signal amplitude, Solution B showed a significantly smaller upstroke to downstroke velocity ratio when compared to the other conditions.

**Figure 7:**
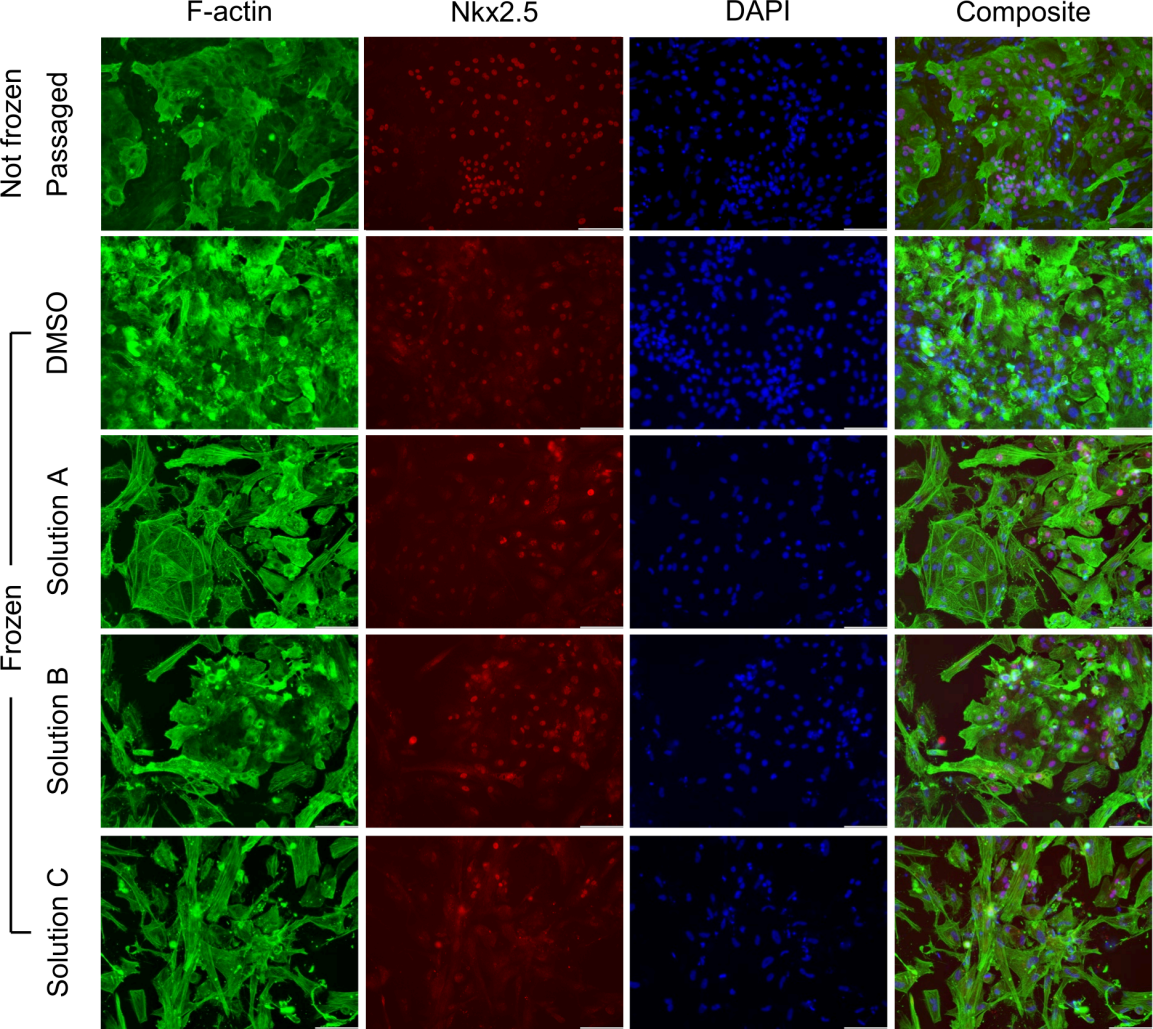
Immunocytochemistry of hiPSC-CMs cryopreserved using DMSO and the optimized DMSO-free CPAs. The first two columns from the left represent the F-actin (green) and Nkx2.5 (red) expression in hiPSC-CMs which were either not frozen or frozen using either DMSO or the optimized DMSO-free CPAs. The third column contains the DAPI counterstain (blue) to detect nuclei. The rightmost column contains the composite image. Scale bar: 100μm.

The final phase of post-thaw assessment involved determining post-thaw immunohistochemistry of the cells to establish that the phenotype was not influenced by the freezing process. Expression of F-actin and Nkx2.5 were used as markers of CM phenotype. It was found that hiPSC-CMs exhibited high expression of F-actin and the Nkx2.5 transcription factor post-thaw, similar to what was observed for unfrozen passaged cells (Fig. 7).

## Discussion

### Biophysical characterization

The studies described in Fig. 1 demonstrated that hiPSC-CMs were larger than the hiPSCs from which they were derived. This difference in size between hiPSCs and differentiated cells is consistent with previous studies which have recorded significant variations in cell size along the differentiation pathway [50, 58]. Though highly variable across sources [59–61], the volume of hiPSC-CMs is usually considered to be around 2000 μm^3^ [51]. Assuming detached hiPSC-CMs in suspension to be perfect spheres would result in a cell diameter of ~ 15.6 μm. This estimate of cell diameter is very close to the diameter recorded in this study (14.86 μm). An osmotically inactive cell volume fraction of 0.4 is comparable to other cell types such as lymphocytes [52], but considerably higher than erythrocytes [53] or hematopoietic progenitor cells [54]. The high osmotically inactive cell volume fraction suggests that the cells will not tolerate high levels of dehydration. This outcome is consistent with the post-thaw osmotic behavior exhibited in Fig. 5. If the cells are less tolerant of dehydration, a higher cooling rate would be beneficial to reduce dehydration of the cells.

While the lack of a noticeable increase in cell volume associated with resuspension in an isotonic medium could be due to the low temporal resolution of the measurements in Fig. 5, numerous studies conducted on the osmotic behavior of rat [66] and guinea pig [67] cardiomyocytes have noted a similar resistance to swelling. In addition, an increase in rabbit cardiomyocyte size due to osmotic stress has been associated with a significant reduction in contractility [68]. Furthermore, metabolically inhibited rat cardiomyocytes have recorded significant sarcolemmal disruption when subjected to osmotic stresses after the resumption of metabolic function [69]. Taken as a whole, there is considerable evidence that hiPSC-CMs could be considered to be osmotically sensitive. This sensitivity may explain poor response to conventional freezing conditions.

### Cryoprotectant Optimization

Previous studies of the freezing behavior of CM have used 10% DMSO as a cryoprotectant and little has been done to look at the influence of varying concentration on post-thaw recovery or function [16, 17, 33]. In this investigation, we used a DE algorithm to optimize the composition of cryopreservation solution. The decision to enhance parameter space resolution was motivated by the complex topology of the space. This enabled us to better understand the impact of minor variations in osmolyte concentrations on the post-thaw recovery of hiPSC-CMs. This decision can be validated by observing the shape of the hiPSC-CM recovery contours, which contain numerous recovery peaks adjacent to regions of low recovery. These variations would have been missed had we chosen a lower osmolyte concentration resolution. The recovery is observed to increase linearly with increase in sugar alcohol concentration. No such clear trend is observed for sugar or amino acid concentrations, and specific concentration combinations yield the recovery hotspots observed in the contours. We have demonstrated in studies that combinations of sugars, sugar alcohols and amino acids form Naturally-occurring Deep Eutectic Systems (NADES) at specific molar ratios [39, 40]. It is possible that the islands of high recovery correspond to NADES formation both inside and outside the cell.

### Influence of freezing parameters

As expected, the cooling rate and nucleation temperature of the freezing process had a significant effect on the post-thaw recovery. In contrast to other cell types, we did not observe an inverted U-shaped curve predicted by Mazur’s two-factor hypothesis [34, 63], according to which both high and low cooling rates correspond to low post-thaw recoveries with an optimum cooling rate in between [63–65]. Instead, as shown in Fig. 3A, we found that a moderate cooling rate of 3°C/min corresponded to worse recovery when compared to higher or lower cooling rates of 5°C/min or 1°C/min. The low temperature Raman spectroscopy did not demonstrate any significant intracellular ice formation at any of the three cooling rates tested. Hence, it is unlikely that the observed outcome reflected the influence of intracellular ice formation. One explanation is that there is one (or more) mechanism of damage that is cooling-rate-dependent but different than the two-factor explanation used for more cell types. If the cell membrane undergoes specific phase transitions during cooling, ‘slow’ cooling could avoid membrane leakage and ‘rapid’ cooling would avoid excessive dehydration, which could be also damaging. We have observed a similar cooling rate dependence for freezing with Natural Killer cells [57] which are also sensitive to conventional freezing protocols [41].

The low temperature Raman spectroscopy gives us additional insights into the freezing response of the cells. We have demonstrated that for different cell types, the partitioning of solute is an important cell response to the freezing environment [29, 44, 62]. A partitioning ratio closer to 1 in Jurkat cells correlates with a higher recovery rate across varying cooling rates and DMSO concentrations [44]. A similar outcome was observed for cardiomyocytes, with the cooling rate of 5°C/min having the highest recovery as well as a partitioning ratio closest to 1. This suggests that the average concentration of DMSO in the extracellular space was nearly equal to the average concentration of DMSO in the intracellular space for a cooling rate of 5°C/min. For a cooling rate of 3°C/min, the partitioning ratio was roughly 0.8. This indicated that the concentration of DMSO inside the cell is greater than the outside. The transport of DMSO across the cell membrane at this particular cooling rate differs from that observed at both higher and lower cooling rates.

The effect of nucleation temperature on the post-thaw recovery was similarly unexpected. It has been known for several decades that lower nucleation temperatures result in a higher fraction of cells that form intracellular ice, a lethal event [38]. We observed that using a lower nucleation temperature of −8°C for hiPSC-CMs yielded higher post-thaw recoveries when compared to −4°C or −6°C. This outcome is consistent with the observation that hiPSC-CMs could be highly sensitive to excessive dehydration. Nucleation at a higher temperature would result in greater cellular dehydration than nucleation at a lower temperature [49], leading to lower post-thaw recovery.

### Post-thaw characterization

Results of post-thaw calcium transient studies of hiPSC-CMs in the literature have been mixed. While some sources report no difference in the calcium handling of hiPSC-CMs post-thaw [42], others have shown a significant impairment in calcium handling post-thaw for hiPSC-CMs derived from specific hiPSC cell lines [17]. In this study, the frozen and thawed hiPSC-CMs demonstrated the ability to attach post-thaw and exhibit calcium transients. It was observed that DMSO-free Solution A and Solution B recorded significantly higher recovery compared to DMSO. Furthermore, measuring the ratio of upstroke to downstroke velocity revealed that both Solution A and DMSO had similarly-shaped calcium transient traces compared to the unfrozen re-plated control. This suggests that the calcium handling of these conditions was not affected by the preservation process. In contrast, Solution B, which contains double the sugar concentration of Solution A, exhibited a significantly smaller upstroke to downstroke velocity ratio when compared to the other conditions. This signaled the possibility of impaired calcium ion intake necessary to initiate the contraction of hiPSC-CMs. A possible explanation for this observation could be that increasing sugar levels in the DMSO-free CPA might correlate to worse attachment and post-thaw function, despite reporting equivalent post-thaw recoveries. Alternatively, similar to the post-thaw recovery contours obtained from the DE algorithm results, there might be specific combinations of sugar and amino acid concentrations that might lead to better post-thaw function. Post-thaw immunophenotyping demonstrated that the cells still expressed CM-specific markers post-thaw. This is in line with the findings from other studies which report post-thaw hiPSC-CMs exhibiting an intact sarcomeric structure and maintaining similar expression of cardiac markers [18, 42].

Despite this, it was observed that the cells that were cryopreserved exhibited variations in intensities of the expressed markers, which could signal the possibility of heterogeneity in the post-thaw population. Additional studies could further examine whether specific subpopulations were enriched or depleted due to a differing response to the freezing protocol. Furthermore, future studies could test the freezing response of hiPSC-CMs derived from other cell lines to our freezing protocol. This would help rule out any variability in outcomes across cell lines. Methods to address the osmotic fragility of hiPSC-CMs, such as introducing a hold step post-thaw before resuspension in the culture medium to allow the cells to regain their mechanical resistance [69] could be tested. Moreover, the effect of other supplements that are conventionally added to cryoprotective media [43] to improve outcomes could be explored. Lastly, in addition to the immediate post-thaw recovery and short-term function analyzed in this study, long-term post-thaw characterization would need to be performed to assess the suitability of a cryopreservation protocol for therapeutic applications [70].

## Conclusions

This study demonstrated that hiPSC-CMs exhibited complex osmotic behavior with a high osmotically inactive cell volume fraction and sensitivity to osmotic stress post-thaw. Combinations of sugars, sugar alcohols and amino acids were able to preserve the cells at high levels of post-thaw recovery. Rapid cooling rates resulted in high levels of post-thaw recovery and improved post-thaw function. Further studies can be conducted to enhance performance and improve post-thaw outcomes. This is critical, as the effective DMSO-free cryopreservation of hiPSC-CMs would greatly accelerate the development of drug discovery and therapeutic applications of hiPSC-CMs.

## Figures and Tables

**Table 1:** Significant peaks of the Raman spectra

Substance	Wavenumber	Assignments
Proteins, lipids	1620–1700	Amide I and C = C stretching [45]
Ice	3087–3162	O-H stretching [46]
DMSO	648–698	Symmetric C-S stretching [47]
oMb	1570–1600	Redox and spin state of heme Fe [48]

## Data Availability

All data generated or analyzed during this study are included in this published article [and its supplementary information files].

## Abbreviations

hiPSC: Human-induced pluripotent stem cell
hiPSC-CM: Human-induced pluripotent stem cell-derived cardiomyocyte
DMSO: Dimethyl sulfoxide
CPA: Cryoprotectant or cryoprotective agent
DE: Differential evolution
IHD: Ischemic heart diseases
RPMI minus: RPMI/B27 medium minus insulin
RPMI+: RPMI/B27 medium
RPMI20: RPMI/B27 medium with 20% fetal bovine serum
CR: Cooling rate
T_NUC_: Nucleation temperature
AO/PI: Acridine orange / propidium iodide
oMb: oxymyoglobin
P: Partitioning ratio
Fluo-4 AM: Fluo-4 acetoxymethyl ester
<F_max_>: Averaged maximum intensity of calcium transient peak
<F_min_>: Averaged minimum intensity of calcium transient peak
F: Difference between <F_max_> and <F_min_>
F_0_: :<F_min_>
NADES: Naturally-occurring deep eutectic systems
